# Amorphous-Amorphous Phase Separation in API/Polymer Formulations

**DOI:** 10.3390/molecules22020296

**Published:** 2017-02-15

**Authors:** Christian Luebbert, Fabian Huxoll, Gabriele Sadowski

**Affiliations:** TU Dortmund, Department of Biochemical and Chemical Engineering, Laboratory of Thermodynamics, Emil-Figge-Str. 70, D-44227 Dortmund, Germany; christian.luebbert@bci.tu-dortmund.de (C.L.); fabian.huxoll@bci.tu-dortmund.de (F.H.)

**Keywords:** poorly water-soluble drug, amorphous solid dispersion, PLGA, thermodynamic model, phase behavior, amorphous-amorphous phase separation

## Abstract

The long-term stability of pharmaceutical formulations of poorly-soluble drugs in polymers determines their bioavailability and therapeutic applicability. However, these formulations do not only often tend to crystallize during storage, but also tend to undergo unwanted amorphous-amorphous phase separations (APS). Whereas the crystallization behavior of APIs in polymers has been measured and modeled during the last years, the APS phenomenon is still poorly understood. In this study, the crystallization behavior, APS, and glass-transition temperatures formulations of ibuprofen and felodipine in polymeric PLGA excipients exhibiting different ratios of lactic acid and glycolic acid monomers in the PLGA chain were investigated by means of hot-stage microscopy and DSC. APS and recrystallization was observed in ibuprofen/PLGA formulations, while only recrystallization occurred in felodipine/PLGA formulations. Based on a successful modeling of the crystallization behavior using the Perturbed-Chain Statistical Associating Fluid Theory (PC-SAFT), the occurrence of APS was predicted in agreement with experimental findings.

## 1. Introduction

Enhancing the solubility and dissolution behavior of poorly-water-soluble active pharmaceutical ingredients (APIs) is a key challenge in the development of new formulations. A successful strategy along this line are amorphous solid dispersions (ASDs) where the APIs are molecularly dissolved in a suitable polymer carrier and thus converted into an amorphous state [1,2]. Several tablet formulations based on this technology have been recently developed [3]. Particularly, polymeric carriers of poly (dl-lactic-co-glycolic acids) (PLGAs) are considered as particularly promising for a variety of medical applications like prolonged release tablets or for depot formulations [4].

**Figure 1:**
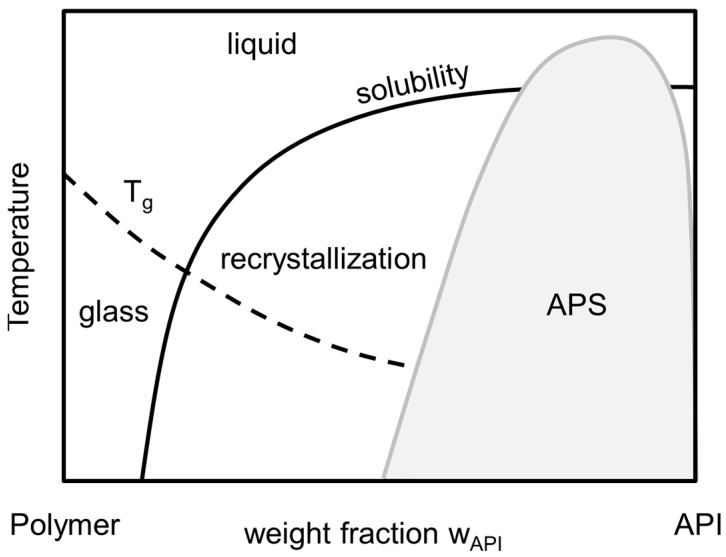
Schematic phase diagram of a API/polymer formulation with solubility line (black line), amorphous-amorphous phase separation (APS) (gray line) and glass-transition temperature (dashed line). Recrystallization occurs at temperatures and compositions below the solubility line. In addition, APS occurs in the light gray region.

The long-term stability of these ASDs is determined by their thermodynamic phase behavior as it determines the maximum API weight fraction of the formulation (*w_API_*) at which it is long-term (i.e., thermodynamically) stable against API recrystallization as well as amorphous-amorphous phase separation (APS) [2]. The thermodynamic phase behavior of an API/polymer formulation is schematically depicted in the phase diagram, shown in Figure 1. Here, the solubility line indicates the composition/temperature range above which an ASD is thermodynamically stable against crystallization: for a given temperature, ASDs are stable against crystallization as long as their composition is left of the solubility line. In addition, the APS region in Figure 1 corresponds to compositions/temperatures where the formation of two liquid (amorphous) phases with different compositions of API and polymer is expected. From thermodynamic point of view, this is a liquid-liquid demixing. This immiscibility is—like crystallization—highly unwanted as it causes heterogeneities resulting in insufficient uniformity of API content in the formulation. For a given temperature, the corresponding concentrations of the two liquid phases can be found on the left and right branches of the APS curve. At conditions below both, the APS line as well as the solubility line, APS can be followed or accompanied by recrystallization, which is not considered here. 

The glass-transition temperature (*T_g_*) depicts the transition temperature from the super-cooled, glassy state of the formulation to its the liquid state. It can be correlated for using empirical approaches, e.g., the Gordon-Taylor-Equation [5] or its modification by Kwei [6]. Although the glass-transition temperature does not mark a thermodynamic phase transition, it indicates a drastic change in mobility and viscosity which usually dramatically decreases the rate of crystallization and/or phase separation and therewith at least kinetically stabilizes ASDs even at thermodynamically-metastable conditions.

Formulations which undergo APS usually show two distinguishable glass-transition temperatures as long as the pure component glass-transition temperatures differ to a certain extend [7]. The presence of two glass-transition temperatures is therefore considered as the main qualitative proof for the immiscibility of API/polymer formulations [8], polymer blends [9,10,11] and amorphous mixtures of APIs and small-molecule excipients, e.g., indomethacin/citric acid [12]. However, due to the high viscosity of the formulations, demixing may take very long time and therefore the quantitative analysis of APS and determining the equilibrium compositions of the two amorphous phases is quite challenging. 

Instead, cloud-point measurements have been used to evaluate APS of polymer (API-free) systems. Koningsveld cooled polymer/solvent mixtures of certain feed compositions until turbidity was detected visually [13], which is the proof of reaching the APS region in the phase diagram. Immiscibility of solvent-free and API-free polymer blends has also been qualitatively evaluated by atomic force microscopy [14], X-ray powder diffraction [15], and micro Raman mapping [11]. Purohit and Taylor applied atomic-force microscopy combined with nanoscale infrared imaging to qualitatively evaluate the immiscibility in the system itraconazole/hydroxypropyl methylcellulose [16]. 

However, none of these studies quantitatively determined the API concentration in the two APS phases as a function of temperature nor even focused on the prediction of APS. For several systems, even contradictory information exists about the existence of APS. Yuan et al. experimentally found an APS in quench-cooled formulations of nifedipine and PVP [17], which was also predicted by Donnelly et al. [18] applying the Flory-Huggins Theory (FHT [19]), although earlier studies [20,21] found complete miscibility of nifedipine and PVP. Thus, there is still an urgent need for reliably determining APS in API/polymer systems experimentally and for theoretical methods being able to correctly predict APS. Whereas the solubility of crystalline APIs was already modeled using adequate thermodynamic models, e.g., FHT [22,23,24], the Perturbed-Chain Statistical Associating Fluid Theory (PC-SAFT) [25] or empirical approaches [26], thermodynamic modeling of APS is quite rare. Lin and Huang [7] experimentally investigated the APS in the system felodipine (FEL) and poly (acrylic acid) which they qualitatively modeled using FHT and a χ-interaction parameter fitted to the crystalline FEL solubility in poly(acrylic acid) obtained from DSC measurements. However, they considered their calculated phase diagram only as a ‘rough draft’ as FHT is a very simplified thermodynamic model for which already Flory [19] pointed out that it is unable to correctly describe demixing phenomena (like APS).

Therefore, in this work the Perturbed-Chain Statistical Associating Fluid Theory (PC-SAFT) [27] was used instead to thermodynamically model solubility and APS in API/polymer formulations. PC-SAFT has already been applied to model the solubility of APIs in organic solvents [28] and the dissolution of APIs in aqueous media [29,30,31,32]. Moreover, it has been shown that PC-SAFT is suitable for describing the solubility of APIs in water-free and humid ASDs [25,33,34,35]. Kiesow et al. successfully predicted the liquid-liquid demixing (oiling out) during API crystallization processes from solvents and could show that PC-SAFT is able to simultaneously model both, solubility and APS (liquid-liquid demixing) in pharmaceutical systems [36].

**Figure 2:**
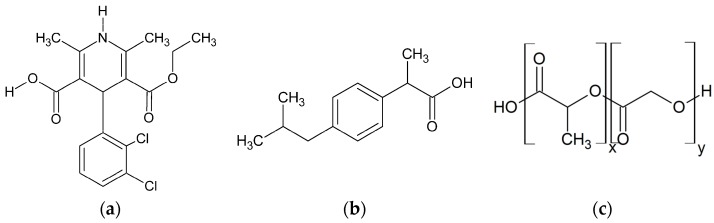
Chemical structures of (**a**) felodipine (FEL); (**b**) ibuprofen (IBU); and (**c**) poly(lactic-co-glycolic acid)s (PLGA) with different molar ratios of the monomers (dl)-lactic acid (x) and glycolic acid (y).

This study investigates the phase behavior of ibuprofen (IBU) and FEL formulations in PLGA-polymers with different molar ratios of (dl)-lactic acid (LA):glycolic acid (GA)-monomers (Figure 2).

The solubility of the crystalline APIs was determined by measuring the solubility temperature of formulations with different API weight fractions via DSC [20]. APS was detected qualitatively by hot-stage microscopy (HSM) and the detection of two glass-transition temperatures in DSC measurements. The experimentally-obtained information about APS was then compared to the PC-SAFT predictions.

## 2. Results and Discussion

### 2.1. Results of DSC Measurements

**Figure 3:**
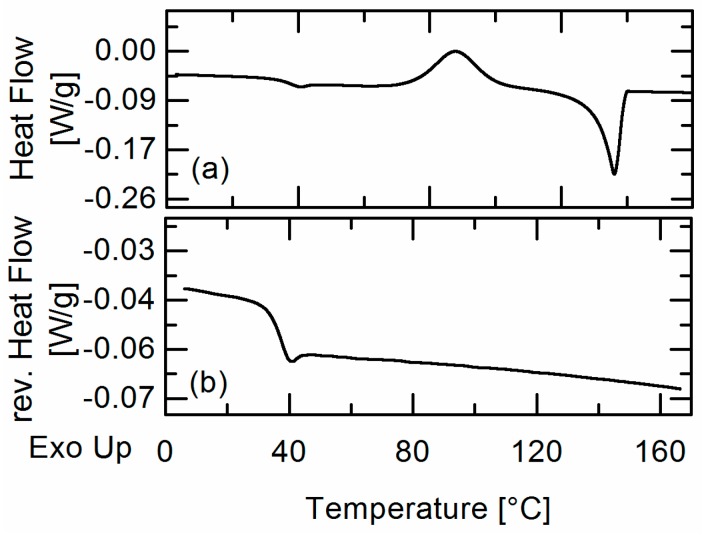
DSC heat flow signal of a homogenous FEL/Resomer^®^ RG 752 S formulation (*w_FEL_* = 0.6) in the first (**a**) and DSC reversing heat flow signal in the second (**b**) heating ramp.

Figure 3 shows as example the obtained DSC heat-flow signal of a formulation composed of FEL and Resomer^®^ RG 752 S with a FEL weight fraction of *w_FEL_* = 0.6. In the heat-flow signal of the first heating ramp (Figure 3a), a glass-transition followed by recrystallization and melting was observed while in the second heating ramp (Figure 3b), only one glass transition (*T_g_* = 37.6 °C) was found for all FEL formulations. They were thus considered to be completely miscible.

**Figure 4:**
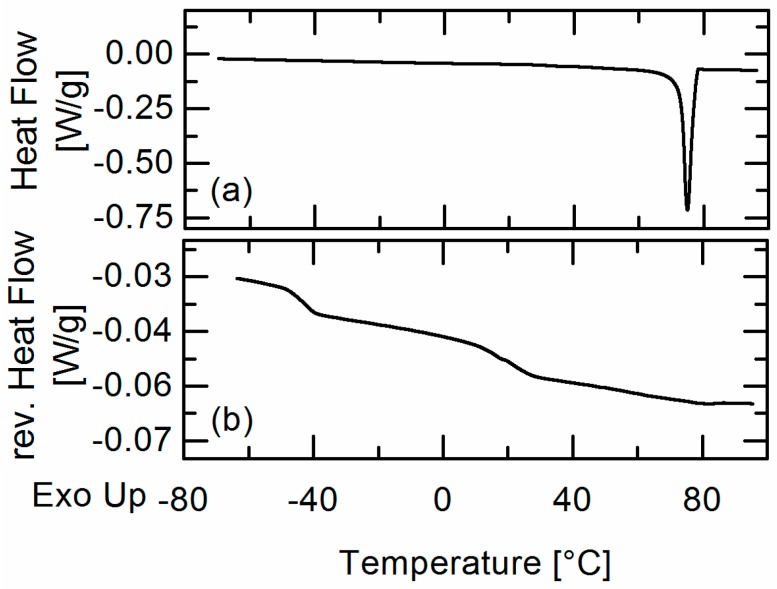
DSC heat flow signal of a IBU/ Resomer^®^ RG 752 S formulation (*w_IBU_* = 0.6) in the first (**a**) and DSC reversing heat flow signal in the second (**b**) heating ramp.

Similar to the FEL formulations, an endothermic melting event was detected in the first heating ramp of IBU formulations (Figure 4a), whereas several formulations (e.g., IBU/Resomer^®^ RG 752 S with an IBU content of *w_IBU_* = 0.6) showed two clearly-distinguishable glass transitions during the second heating ramp as to be seen in Figure 4b.

For those formulations, the presence of an APS in the investigated temperature range was concluded. Whereas the first glass transition in these formulations was detected at a temperature of about *T_g_*_,1_ = −42.3 °C corresponding to the glass-transition temperature of pure IBU, the second glass transition occurred at temperatures between 28.7 °C and 33.4 °C for Resomer^®^ RG 502, between 16.3 °C and 22.4 °C for Resomer^®^ RG 752 S, and between 2.3 °C and 20.3 °C for Resomer^®^ R 202 S (Table 1).

### 2.2. Results of Hot-Stage Microscopy

In order to verify the APS for the investigated systems with a second experimental method and to elucidate the events observed in the DSC curves, HSM measurements were performed as described in Section 5.4. For none of the FEL formulations, APS was observed. Upon heating from room temperature to 150 °C, crystalline FEL melted forming a homogeneous liquid PLGA/FEL phase which remained homogenous while cooling down to room temperature (results not included in this publication).

**Figure 5:**
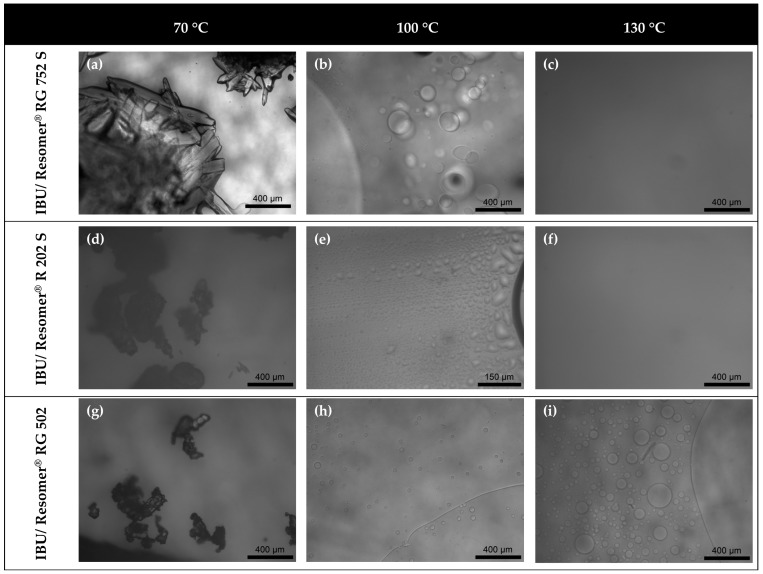
HSM images of IBU/PLGA-formulations with *w_IBU_* = 0.7 at 70 °C (**a**,**d**,**g**), 100 °C (**b**,**e**,**h**) and 130 °C (**c**,**f**,**i**) showing the melting of IBU crystals followed by APS.

HSM images taken from IBU formulations at a temperature below the melting temperature of pure IBU (70 °C) and two images at temperatures above the melting temperature of IBU (100 °C and 130 °C) are depicted in Figure 5. Images were taken from formulations with the three investigated PLGAs Resomer^®^ R 202 S, Resomer^®^ RG 752 S and Resomer^®^ RG 502 all having the same weight fraction of IBU *w_IBU_* = 0.7.

As can be seen from the images of the IBU/Resomer^®^ RG 752 S formulation (Figure 5a), IBU crystals were observed at a temperature of 70 °C. Upon further heating above the melting temperature of IBU (77.09 °C) to a temperature of 100 °C, IBU crystals melted and droplets were observed (Figure 5b). At this temperature, the formulation demixed into two liquid phases. Upon heating up to a temperature of 130 °C, the droplets coalesced resulting in one homogenous phase (Figure 5c).

IBU crystals were found also for the other investigated copolymer formulations at a temperature of 70 °C (Figure 5d,g). Moreover, the subsequent melting (dissolution) of IBU in the PLGAs upon heating was observed for all PLGA types leading to two liquid phases (droplets of one phase in the other) at 100 °C in all cases (Figure 5e,h). The droplets coalesced upon heating to a temperature of 130 °C for the copolymers Resomer^®^ R 202 S and Resomer^®^ RG 752 S resulting in one homogenous phase in the microscopic images (Figure 5c,f), whereas APS was observed in the Resomer^®^ RG 502-formulation over the whole investigated temperature range up to 130 °C (Figure 5i).

### 2.3. Phase Behaviour of API/Polymer Formulations

#### 2.3.1. FEL/PLGA Formulations

**Figure 6:**
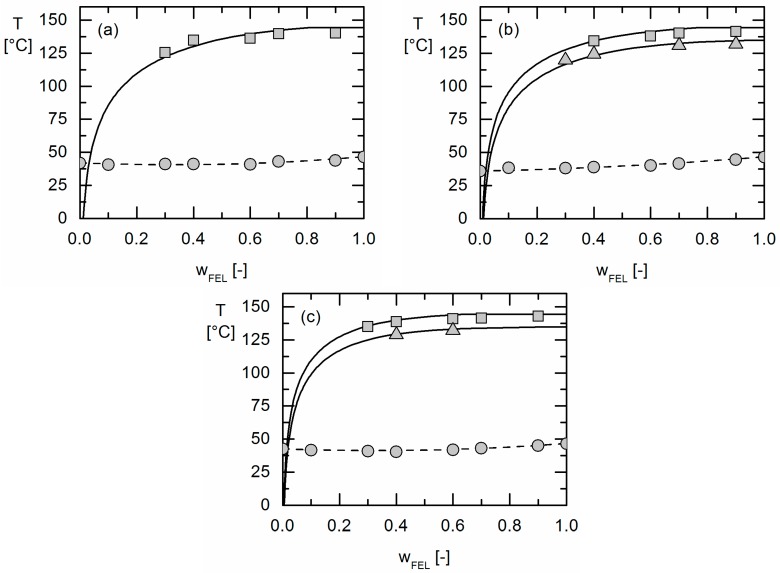
Phase diagrams of FEL/Resomer^®^ R 202 S (**a**); FEL/Resomer^®^ RG 752 S (**b**) and FEL/Resomer^®^ RG 502 (**c**). Symbols are experimental data obtained via DSC measurements in this work. Gray circles are glass-transition temperatures, squares the solubility temperatures of FEL polymorph I and triangles are solubility temperatures of FEL polymorph II. Black lines are the solubility lines as modeled using PC-SAFT, the dashed lines are glass-transition temperatures modeled using the Kwei-Equation.

Glass-transition temperatures and solubility temperatures of FEL in PLGA formulations were investigated by DSC as described in Section 3.1. The results are shown in Figure 6. The error bars of the experimental data are smaller than the symbols and are thus not visible in the diagrams.

The glass-transition temperatures of the formulations were partly found below the glass-transition temperatures of the polymer and FEL. This phenomenon was already observed in earlier studies for this type of polymers [35] and required the application of the Kwei-Equation for an accurate modeling. Using the Kwei-parameters k and q fitted to Equation (6) as summarized in Table 2, the glass-transition temperatures could be modeled with high accuracy (Figure 6).

The PC-SAFT pure-component parameters for FEL were determined by fitting to FEL solubility data in different organic solvents as described by Ruether and Sadowski [28]. They are listed together with the pure-component parameters for IBU, PDLA, PLLA and PGA in Table 3. The FEL solubility in the investigated organic solvents together with the modeling results can be found in the Supplementary Materials of this publication.

The PC-SAFT binary interaction parameters *k_ij_* between the unlike copolymer segments were set to zero [33,35]. Moreover, the *k_ij_* parameters between PDLA and PLLA at the one hand and the APIs at the other hand were assumed to be the identical and fitted to the solubility of IBU in Resomer^®^ R 202 S, which only comprises PDLA and PLLA monomers (Figure 6a). As the PGA homopolymer is semi-crystalline and does not dissolve APIs, the *k_ij_* between PGA and API was fitted to the experimentally-determined phase behaviour of the copolymer with the highest amount of PGA (Resomer^®^ RG 502, Figure 6c). The binary interaction parameters used for the PC-SAFT modeling are summarized in Table 3.

Based on these parameters which were fitted to the FEL solubility in the homopolymers PLLA, PDLA and PGA only, the FEL solubility in Resomer^®^ RG 752 S could be predicted without fitting any additional parameters (Figure 6b).

It is worth mentioning that the PC-SAFT parameters for the two FEL polymorphs are identical as PC-SAFT models the FEL activity coefficient in the liquid phase which depends on the properties of the liquid only and is not affected by the solid it is in equilibrium with. Thus, the binary interaction parameter between FEL and the respective homopolymers PLLA, PDLA and PGA was fitted to the solubility of polymorph I only and was then used to predict the solubility of polymorph II using the different melting properties of the latter as given in Table 4. As can be seen from the phase diagrams in Figure 6a–c, also the experimentally-observed solubility temperatures of polymorph II are in very good agreement with the predicted ones.

In order to evaluate the PC-SAFT modeling, the maximum absolute deviation (*MAD*) and the average absolute deviation (*AAD*) of the modeled solubility of the crystalline FEL from the experimentally-determined one were determined according to Equations (1) and (2):

(1) $$MAD=\underset{i=1,n_{exp}}{\max}\left|T_{calc,i}-T_{exp,i}\right|$$

(2) $$AAD=\frac{1}{n_{exp}}\cdot \overset{n_{exp}}{\underset{i=1}{\sum }}\left|T_{calc,i}-T_{exp,i}\right|$$

*MAD* and *AAD* values are given in Table 5 for all investigated FEL formulations. It can be seen that solubility of crystalline FEL in all PLGA as well as the glass-transition temperatures can be described with high accuracy. 

Using the PC-SAFT parameters fitted to pure-component data and solubility data of FEL in PLGA only, blind APS predictions were formed according Equations (4) and (5). As a result, it was predicted that the FEL formulations considered in this work do not undergo APS. These predictions were perfectly validated by the HSM experiments where APS was not observed either.

#### 2.3.2. IBU/PLGA Formulations

**Figure 7:**
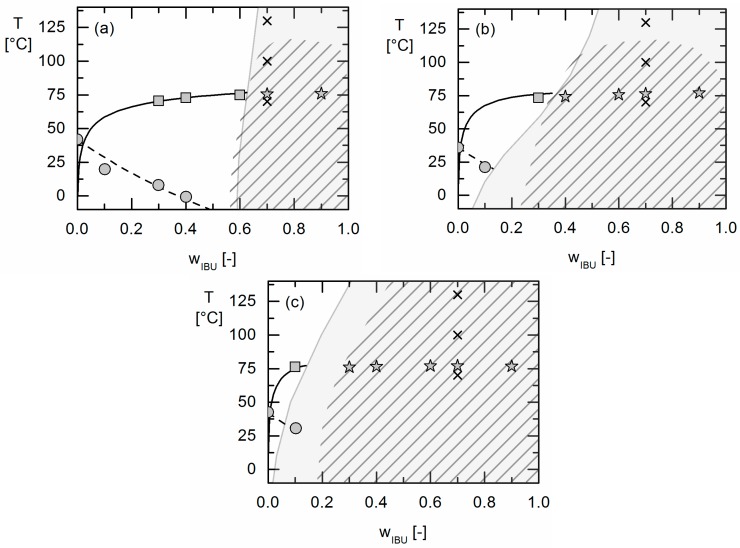
Phase diagrams of the formulations IBU/Resomer^®^ R 202 S (**a**); IBU/ Resomer^®^ RG 752 S (**b**); IBU/Resomer^®^ RG 502 (**c**). Symbols are experimental data obtained in this work. Gray circles are glass-transition temperatures, squares are solubility temperatures of crystalline IBU and stars are solubility temperatures of IBU in the APS region (all determined via DSC measurements). Black lines are the solubility lines as modeled using PC-SAFT, the dashed lines are glass-transition temperatures modeled using the Kwei-Equation. Gray-shaded areas are PC-SAFT predicted APS regions. Hatched areas indicate conditions where APS is expected form HSM and DSC (two glass transitions). The crosses mark the points where images were taken with HSM (Figure 5).

The phase behavior of IBU/PLGA formulations was determined via DSC measurements (Section 2.1) and HSM imaging (Section 2.2). The phase diagrams resulting from these measurements are shown in Figure 7.

The IBU solubility temperatures in the three investigated polymers measured in the first heating ramp of the DSC are represented by gray squares in the phase diagrams (Figure 7). Stars indicate the transition from a formulation containing IBU crystals to a demixed formulation with two amorphous phases (observed in the HSM experiments). The DSC heat flow signals for this transition did not differ from those of a crystalline sample forming a homogenous phase after melting (Figure 3a). The melting temperatures in the APS region were quite similar to the melting temperatures of pure IBU and almost no depression in melting point was detected. Measured glass-transition temperatures for (homogenous) formulations which showed only one glass transition are indicated by gray circles in Figure 7. At room temperature, the IBU solubility in PLGA was found to be very low irrespective of the (DL) LA:GA ratio, which is consistent with earlier studies [35]. Increasing the GA content in the copolymer from 0 mol % (Resomer^®^ R 202S) to 50 mol % (Resomer^®^ RG 502) only slightly influences the solubility of IBU in the copolymers, whereas it significantly influences the APS.

The exact size of the APS region could not be experimentally determined. Karavas et al. [39] proposed to calculate the concentrations of IBU in the two phases of the demixed formulation by relating the two glass-transition temperatures to apparent phase compositions. However, temperature changes during a DSC measurement and thus the thermodynamic equilibrium and therewith the compositions of the two demixed phases might also change during a heating ramp. The correlation of concentrations and glass-transition temperatures of the phases is therefore considered to be error prone and was not applied here. Instead, the APS region was estimated qualitatively based on the experiments mentioned above: formulation compositions showing two glass-transition temperatures in DSC experiments (which determined the width of the immiscibility region) and HSM images (which were used to determine the upper limit of the APS region). The result is indicated by the hatched area in Figure 7.

The IBU concentration in the polymer-rich phase was found to be lowest in Resomer^®^ RG 502 formulations and highest in Resomer^®^ R 202S formulations (left-hand side of the hatched areas in Figure 7). The APS region was thus found to be broadest for the Resomer^®^ RG 502 systems (Figure 7c). As the glass-transition temperature of the IBU-rich phase was almost the same as for pure IBU, this phase can be assumed to contain almost no Resomer^®^ but almost pure IBU (right-hand side of the phase diagrams shown in Figure 7).

Solubility and glass-transition temperatures of IBU/PLGA formulations were modeled based on the DSC measurements. The binary PC-SAFT interaction parameters were fitted as described above to the solubility of IBU in Resomer^®^ R 202 S and Resomer^®^ R 502 (Table 3). The glass-transition temperature was modeled using the Kwei-Equation by calculating the *k*-parameter from Equation (7) and fitting the q-value to the measured glass-transition data (Table 2). Glass-transition temperatures and IBU solubilities were not calculated within the APS region as the latter is the thermodynamically-stable one and thus calculations of both are not valid there. Experimental data and modeling results are compared in Figure 7. The *MAD* and *AAD* values for the modeling compared to the experimental data are shown in Table 6. For the calculation of *MAD* and *AAD*, only results of homogenous formulations were considered, the results from the APS region were neglected.

The modeling of the glass-transition with the empirical Kwei-Equation (Equation (6)) is found to be in very good accordance to the experiments (see Table 6). Moreover, as can be seen from Table 6 and Figure 7, PC-SAFT is capable to model the solubility of IBU in the PLGAs very precisely. Using the binary interaction parameters fitted to IBU solubility data in the Resomer^®^ R 202 S and Resomer^®^ RG 502, the APS in the three IBU/Resomer^®^ formulations was then blindly predicted applying Equations (4) and (5) without any additional parameter fitting and without even assuming that an APS exists. As a result, APS was predicted for all IBU/Resomer^®^ formulations considered in this work. As can be seen from Figure 7, APS was indeed also experimentally observed for all systems. Moreover, also the quantitative agreement of predicted and experimentally-observed APS is remarkable, particularly in the temperature range relevant for pharmaceutical formulations. 

**Figure 8:**
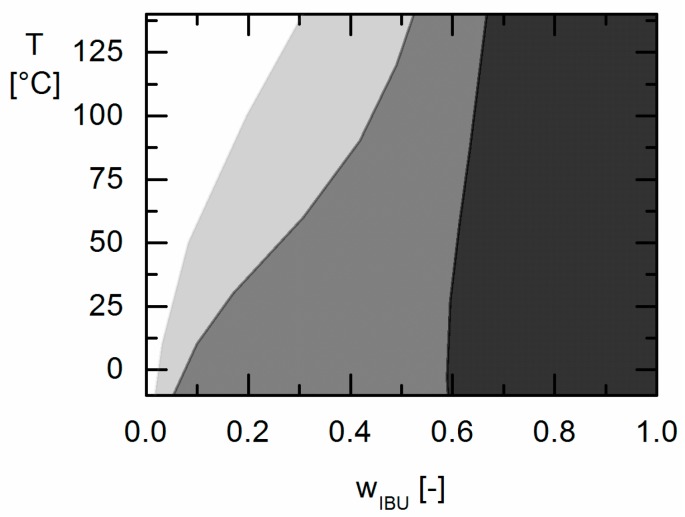
Influence of the copolymer composition on the APS region for IBU/PLGA formulations predicted via PC-SAFT. Predicted APS for a GA-content of 0 mol % (Resomer^®^ R 202 S) is black shaded. Predicted APS for 25 mol % GA (Resomer^®^ RG 752 S) is gray shaded and predicted APS for 50 mol % GA (Resomer^®^ RG 502) is shaded light gray.

For a more detailed view, the APS regions of the three IBU/Resomer^®^ formulations are shown in Figure 8 to study the influence of the copolymer composition on size and shape of the APS region.

The polymer Resomer^®^ R 202 S containing only (dl)LA monomers showed the narrowest APS region. At 25 °C, APS occurs only at IBU weight fractions above *w_IBU_* = 0.595. By increasing the amount of GA in the copolymer to 25 mol % in Resomer^®^ RG 752 S, the APS region gets broader. For this polymer, APS is expected to occur in formulations with an IBU weight fraction higher than 0.176. In Resomer^®^ RG 502 with 50 mol % GA, the APS region is biggest and IBU formulations with API contents greater than *w_IBU_* = 0.047 might undergo phase separation. Thus, the width of the APS is strongly influenced by copolymer composition in the whole temperature range. In order to avoid APS during processing and storage, PLGAs with low GA-content should be preferred.

## 3. Modeling

### 3.1. Solubility of Crystalline APIs in Polymers

The mole fraction solubility $x_{API}^{L}$ of an API in a liquid (amorphous) API/polymer phase (*L*) is calculated from Equation (3) applying the equilibrium condition that at temperature *T* the chemical potential of the *API* equals in both, the liquid and the solid (*S*) phase:

(3) $$x_{API}^{L}=\;\frac{1}{\gamma_{API}^{L}}\exp\left\{-\frac{\Delta h_{API}^{SL}}{RT}\left(1-\frac{T}{T_{API}^{SL}}\right)-\frac{\Delta c_{p,API}^{SL}}{R}\left[\ln\left(\frac{T_{API}^{SL}}{T}\right)-\frac{T_{API}^{SL}}{T}+1\right]\right\}$$

$\Delta h_{API}^{SL}$ (J·mol^−1^) is the melting enthalpy, $T_{API}^{SL}$ (K) is the melting temperature and $\Delta c_{p,\;API}^{SL}$ (J·mol^−1^·K^−1^) is the difference in heat capacity of the liquid and solid *API* at the melting temperature. These properties can be determined experimentally (e.g., by DSC measurements). *R* is the ideal gas constant (8.314 J·mol^−1^·K^−1^). The activity coefficient $\gamma_{API}^{L}$ of the *API* in the liquid phase accounts for the thermodynamic non-ideality of the *API* in the polymer matrix. It is calculated in this study using the thermodynamic model PC-SAFT [27].

### 3.2. Amorphous-Amorphous Phase Separation (APS)

During APS, a mixture of polymer and *API* demixes into two liquid (amorphous) phases leading to a polymer-rich phase and an API-rich phase (denoted as *L1* and *L2*, respectively) with mole-fraction compositions of polymer and *API* $x_{API}^{L1}$ and $x_{polymer}^{L1}$ in phase *L1* and of $x_{API}^{L2}$ and $x_{polymer}^{L2}$ in phase *L2*. The chemical potentials of both, *API* and polymer, must be the same in the two liquid phases leading to Equations (4) and (5) which have to be solved simultaneously:

(4) $$x_{API}^{L1}\gamma_{API}^{L1}=x_{API}^{L2}\gamma_{API}^{L2}$$

(5) $$x_{polymer}^{L1}\gamma_{polymer}^{L1}=x_{polymer}^{L2}\gamma_{polymer}^{L2}$$

The activity coefficients of *API* ($\gamma_{API}$) and polymer ($\gamma_{polymer}$) again account for the thermodynamic non-idealities of the two components in the ASD and were, like for the solubility calculations, obtained via PC-SAFT. 

### 3.3. Glass-Transition Temperature

The glass-transition temperature of the API/polymer formulations was modeled using the Kwei-Equation [6] as function of the *API* weight fraction in the formulation $w_{API}$ ($w_{polymer}=1-w_{API}$):

(6) $$T_{g}=\frac{w_{API}\;T_{g,API}+k\;w_{polymer}\;T_{g,polymer}}{w_{API}+k\;w_{polymer}}+q\;w_{API}\;w_{polymer}$$

The parameter *q* was fitted to measured glass-transition temperatures of the API/polymer formulation. The constant k was estimated from Equation (7):

(7) $$k=\frac{\rho_{API}\;T_{g,API}}{\rho_{polymer}\;T_{g,polymer}}$$

using the densities of the polymer $\rho_{polymer}$ and the amorphous API $\rho_{API}$ as well as the glass-transition temperatures (K) of the pure components.

### 3.4. PC-SAFT

PC-SAFT [27] is a thermodynamic model for the Helmholtz energy of a system and can be used to calculate any other thermodynamic property of interest, e.g., activity coefficients of system components. In this model, molecules like APIs or polymers are considered as chains of $m^{seg}$ spherical segments with a diameter σ. These two geometrical parameters and one dispersion-energy parameter $u/k_{B}$ ($k_{B}$ being the Boltzmann constant) accounting for van der Waals interactions are required to characterize non-associating molecules (e.g., PLGAs). Associating molecules like APIs are components which are able to interact with other molecules via formation of hydrogen bonds. This is taken into account in PC-SAFT by the number of $N^{assoc}$ association sites for each associating molecule (usually derived from the molecular structure) and the association volume $k_{hb}^{A_{i}B_{i}}$ characterizing the range of the association potential as well as the association energy of $\varepsilon_{hb}^{A_{i}B_{i}}/k_{B}$ characterizing the association strength of a molecule.

Using these pure-component parameters, the residual Helmholtz energy $a^{res}$ is calculated as sum of different contributions accounting for the hard-chain repulsion $a^{hc}$, van-der-Waals attraction (dispersion) $a^{disp}$ and association $a^{assoc}$:

(8) $$a^{res}=\;a^{hc}+\;a^{disp}+\;a^{assoc}$$

Modeling of mixtures like investigated in this study further require mixing rules for the parameters of components i and j. The Berthelot-Lorentz combining rules were applied according to Equations (9) and (10) to describe the segment diameter $\sigma_{ij}$ and dispersion energy $u_{ij}$ in the mixture:

(9) $$\sigma_{ij}=\;\frac{1}{2}\;\left(\sigma_{i}+\;\sigma_{j}\right)$$

(10) $$u_{ij}=\left(1-k_{ij}\right)\sqrt{u_{i}u_{j}}$$

The parameter $k_{ij}$ in Equation (10) corrects the dispersion energy in the mixture and was fitted in this work to the solubility of the crystalline API in the polymer. A more detailed summary of the PC-SAFT equations can be obtained from [27].

#### 3.4.1. Calculation of Activity Coefficients from PC-SAFT

The activity coefficient $\gamma_{i}^{L}$ of the component i in the liquid is required for calculating the solubility (Equation (3)) and APS (Equations (4) and (5)). It is defined as the ratio of the fugacity coefficient of the component in the mixture $\varphi_{i}^{L}$ and the fugacity coefficient of the pure component $\varphi_{0,i}^{L}$ (= $\varphi_{i}^{L}$ for $x_{i}^{L}\to 1)$ (Equation (11)):

(11) $$\gamma_{i}^{L}=\;\frac{\varphi_{i}^{L}}{\varphi_{0,i}^{L}}$$

The fugacity coefficients can be determined according to Equation (12) from the residual chemical potential ${µ}_{i}^{res}$ and the compressibility factor Z:

(12) $$\ln\varphi_{i}^{L}=\;\frac{{µ}_{i}^{res}}{k_{B}T}-\ln Z$$

The compressibility factor *Z* and the residual chemical potential ${µ}_{i}^{res}$ are calculated from the residual Helmholtz energy $a^{res}$ (obtained from PC-SAFT) according to Equations (13) and (14) with ρ being the density of the system:

(13) $$Z=1+\rho \left[\frac{\partial \left(a^{res}/k_{B}T\right)}{\partial \rho }\right]$$

(14) $$\frac{{µ}_{i}^{res}}{k_{B}T}=\;\frac{a^{res}}{k_{B}T}+Z-1+\left[\frac{\partial \left(a^{res}/k_{B}T\right)}{\partial x_{i}}\right]-\overset{N}{\underset{j=1}{\sum }}\left[x_{j}\left(\frac{\partial \left(a^{res}/k_{B}T\right)}{\partial x_{j}}\right)\right]$$

#### 3.4.2. Modeling the Copolymer PLGA with PC-SAFT

PLGA is a random copolymer consisting of the monomer units DLA, LLA and GA. PC-SAFT allows calculating thermodynamic properties of copolymers only based on the parameters of the respective homopolymers *α* poly(glycolic acid) (PGA), poly (D-lactic acid) (PDLA), and poly(l-lactic acid) PLLA)) [40]. Knowing the homopolymer units which the copolymer consists of, the copolymer composition is characterized by segment fractions $z_{i,\alpha }$ and bond fractions $B_{i\alpha i\beta }$.

The segment fraction is defined as the fraction of segments α ($m_{i,\alpha }^{seg}$) among all segments $m_{i}^{seg}$ (originating from the different monomer units) in the copolymer chain *i*:

(15) $$z_{i,\alpha }=\frac{m_{i,\alpha }^{seg}}{m_{i}^{seg}}$$

whereas the number of *α* segments is obtained as:

(16) $$m_{i,\alpha }^{seg}=\frac{w_{i,\alpha }M_{copol}}{{\left(\frac{m^{seg}}{M}\right)}_{\alpha }}$$

$w_{i,\alpha }$ is the mass fraction of *α* segments in the copolymer *i* and ${\left(\frac{m^{seg}}{M}\right)}_{\alpha }$ is a parameter of homopolymer *α* (see Table 9). $M_{copol}$ is the molar mass of the copolymer. The segment fractions $z_{i,\alpha }$ for the three investigated copolymers were calculated using Equations (15) and (16) and are given in Table 7.

The bond fraction $B_{i\alpha i\beta }$ is the fraction of bonds between two different segment types α and β in the copolymer and depends on the arrangement of segments α and β in the copolymer chain. As already shown by Prudic et al. [35], the bond fractions for PLGA can be obtained using the equations in Table 8, assuming that DLA or LLA segments were not directly bonded to each other in Resomer^®^ RG 502. *n*_PLGA,LLA_, *n*_PLGA,DLA_ and *n*_PLGA,GA_ represent the mole numbers of monomers LLA, DLA and GA in the PLGA.

All calculations (solubility of the crystalline API and APS) were performed using the same set of pure-component parameters summarized in Table 9. 

## 4. Materials

Felodipine (FEL) was purchased as light-yellow form-I polymorph (melting point of form I confirmed by DSC measurements) with a purity of 99.7% from Discovery Fine Chemicals Ltd. (Wimborne, UK). The racemic (*R*,*S*)-IBU with a purity of more than 98% was purchased in crystalline form from TCI Deutschland GmbH (Eschborn, Germany). Poly(lactic-co-glycolic acid)s (PLGAs) with a mole fraction of GA of 25 mol % (Resomer^®^ RG 752 S) and 50 mol % (Resomer^®^ RG 502) and pure PDLLA (Resomer^®^ R 202 S) were obtained from Evonik Pharma Polymers (Darmstadt, Germany). Acetone with a purity of 99.9%, obtained from VWR International GmbH (Darmstadt, Germany) was used to prepare solutions of API and the polymers for spray drying (FEL) and solvent evaporation (IBU).

## 5. Methods

### 5.1. Preparation of Formulations via Spray Drying

For each of the three polymers, six FEL-Formulations with a weight fraction of *w_FEL_* = 0.1, 0.3, 0.4, 0.6, 0.7, 0.9 were prepared by spray drying the API (2 g) and polymer dissolved in acetone (200 mL). Powders were weighted with an accuracy of ±0.1 mg. The lab scale spray dryer B-290 by Büchi Labortechnik GmbH (Essen, Germany) with the inert loop B-295 to condense the solvent from the gas stream was used to prepare all formulations. The inlet temperature of the aspirator was set to 60 °C and nitrogen was used as spray gas for all experiments at a volumetric flow of 550 L/h. The peristaltic pump fed the solution with a pump rate of 8 mL/min into the spray nozzle. The aspirator was set to 100% (approx. aspirator gas flow 35 m^3^/h). Residual solvent was removed from the formulations by storage under vacuum for 24 h prior to further analysis.

### 5.2. Preparation of Formulations via Solvent Evaporation

As the low glass-transition temperature of IBU (−42.3 °C) leads to glass-transition temperatures of the formulations which were below the outlet temperature of the spray drying process, a solvent-evaporation method was chosen to prepare formulations instead. Formulations with the weight fractions *w_IBU_* = 0.1, 0.3, 0.4, 0.6, 0.7, 0.9 were prepared by weighing 2 g API and polymer with an accuracy of 0.1 mg and dissolving it in 20 mL acetone. In a second step, acetone was removed under vacuum for one week until no further loss in weight could be detected. Before use, samples were grounded with a mortar to powder.

### 5.3. Modulated Differential Scanning Calorimetry

All formulations were investigated via two heating ramps by modulated differential scanning calorimetry (DSC) to determine the glass-transition temperature as well as the solubility temperature [42]. Measurements were carried out using a Q2000 DSC by TA Instruments (New Castle, DE, USA) with a RCS90 cooling device. Prior to use, temperature was calibrated against the melting temperature of pure indium and the heat capacity was calibrated with a sapphire standard. During measurements, the DSC cell was purged with a nitrogen flow of 50 mL·min^−1^. Standard pans were filled with 10–20 mg of a formulation and covered with a perforated lid.

IBU formulations were investigated in a temperature range from −60 °C to 90 °C and FEL-formulations in a temperature range from 0 °C to 180 °C. Every sample was heated up with an average heating rate of 2 K·min^−1^ (first heating ramp) to melt partly recrystallized formulations, rapidly cooled down with a rate of 10 K·min^−1^ (cooling ramp) and then heated up again with the initial heating rate (second heating ramp). The heating rate of 2 K·min^−1^ was overlapped by a sinusoidal modulation at a period time of 60 s and an amplitude of 0.318 K·min^−1^ [42]. 

The melting offset temperature was determined from the total heat flow signal in the first ramp. As pointed out by Tao et al., the measured melting temperature depends on the heating rate [23] and was therefore extrapolated using additional measurements at heating rates of 1 K·min^−1^ and 5 K·min^−1^ to a heating rate of 0 K·min^−1^. In the second heating ramp, the glass-transition temperatures of the formulations were determined from the reversing heat flow signal after removal of the thermal history of the samples during the first heating ramp [23]. Solubility and glass-transition temperatures were determined with an accuracy of ±0.141 K.

### 5.4. Hot-Stage Microscopy

To qualitatively investigate the phase behavior of the formulations, they were investigated by HSM at a heating rate of 2 K·min^−1^ in the same temperature range as for the DSC measurements. Experiments were performed on a Linkam THMS600 hot stage (Tadworth, UK) mounted to a Leica DM4000M microscope (Wetzlar, Germany). A sample of 5–10 mg was placed onto the heating block and HSM images were taken in time frames of 60 s.

## 6. Conclusions

In this work, amorphous-amorphous phase separation in API/PLGA formulations was investigated via DSC measurements and HSM. Two glass-transition temperatures in the DSC measurements and visible droplets in the HSM images were detected for IBU/Resomer^®^ systems showing these systems undergo APS in the considered temperature range. In contrast, no APS was found for FEL/Resomer^®^ systems. 

The solubility of crystalline APIs in Resomers^®^ could be modeled precisely using the thermodynamic model PC-SAFT by fitting one binary interaction parameter to measured solubility data. Using these parameters fitted to solubility data only, the presence or absence of APS in these API/polymer formulations was purely predicted using PC-SAFT. As a result, the existence of APS was blindly predicted for the investigated IBU/Resomer^®^ formulations whereas a homogeneous amorphous phase was predicted for the FEL/Resomer^®^ formulations. These predictions were validated by the experimental findings of this work. This is remarkable as melting point depression caused by the Resomers^®^ was very similar for IBU and FEL. Moreover, it was found that formulations with high IBU weight fractions undergo APS more likely than those with small IBU loadings.

Moreover, the influence of different GA contents in the Resomers^®^ on APS formation was predicted in qualitative and even almost quantitative agreement with the experimental data. While the increasing GA content influences the solubility (crystal formation) at room temperature only slightly, it strongly affects the APS region leading to more extended APS regions with increasing GA content in the Resomer^®^ (biggest for Resomer^®^ RG 502). 

Using thermodynamic phase diagrams allows for estimating the processability window of API/PLGA formulations as well its long-term behaviour during storage. Processing formulations with API loadings in the APS region might cause APS already at extruder conditions or after long-term storage (even at dry conditions) resulting in non-homogeneous formulations with undesired release performance and unpredictable bioavailability. By applying thermodynamic modeling using PC-SAFT, APS can be estimated at conditions relevant for processing (above the melting point of the API in the case of extrusion processes) or long-term storage (25 °C and 40 °C).

As a result of this work evidently low the GA contents in the copolymer (as for Resomer^®^ R 202 S) and low IBU contents (e.g., below *w_IBU_* = 0.595 in IBU/Resomer^®^ R 202 S formulations) in IBU/Resomer^®^ prevent APS during processing and/or long-term storage.

## Figures and Tables

**Table 1:** Measured *T_g_* values for IBU formulations.

*w_IBU_*	IBU/Resomer^®^ R 202 S		IBU/Resomer^®^ RG 752 S		IBU/Resomer^®^ RG 502	
	*T*_*g*,1_ (°C)	*T*_*g*,2_ (°C)	*T*_*g*,1_ (°C)	*T*_*g*,2_ (°C)	*T*_*g*,1_ (°C)	*T*_*g*,2_ (°C)
0.3	-	-	−49.6	16.3	−43.2	28.7
0.4	-	-	−49.1	16.8	−43.4	30.5
0.6	−42.6	2.3	−42.8	22.4	−42.7	31.3
0.7	−41.2	19.3	−42.5	16.8	−43.6	30.6
0.9	−41.8	20.3	−42.1	17.5	−42.9	33.4

**Table 2:** Kwei-parameters used for modeling the glass-transition temperatures of the investigated API/polymer systems.

API/Polymer System	*k*	*q*
IBU/Resomer^®^ R 202 S	0.615	0.1
IBU/Resomer^®^ RG 752 S	0.765	−15.9
IBU/Resomer^®^ RG 502	0.731	−12.7
FEL/Resomer^®^ R 202 S	1.039	−13.5
FEL/Resomer^®^ RG 752 S	1.060	−7.0
FEL/Resomer^®^ RG 502	1.037	−12.3

**Table 3:** Binary PC-SAFT interaction parameters between the APIs and homopolymers.

	IBU	FEL
PLLA	0.052	−0.0065
PDLA	0.052	−0.0065
PGA	0.105	0.007

**Table 4:** Liquid densities, *T_g_*’s and melting properties of the substances investigated in this work.

Component	*ρ* (g/cm^3^)	*T_g_* (°C)	*T^SL^* (°C)	∆*h^SL^* (kJ/mol)	$\Delta c_{p,\;API}^{SL}$ (J/mol·K)
FEL (I)	1.28 ^b^	46.6 ^a^	143.70 ^a^	30.83 ^b^	89.87 ^a^
FEL (II)	1.28 *	46.6 *	134.80 ^e^	27.60 ^e^	89.87 *
IBU	1.05 ^b^	−42.3 ^a^	77.09 ^a^	25.50 ^c^	50.30 ^c^
Resomer^®^ R 202 S	1.25 ^f^	41.9 ^a^			
Resomer^®^ RG 752 S	1.25 ^f^	35.8 ^d^			
Resomer^®^ RG 502	1.25 ^f^	42.5 ^a^			

^a^ Measured in this work; ^b^ taken from Marsac et al. [20]; ^c^ taken from Gracin and Rasmuson [37]; ^d^ taken from Prudic et al. [35]; ^e^ taken from Surov et al. [38]; ^f^ information from the supplier; * assumed to be the same for the two polymorphs.

**Table 5:** *MAD* and *AAD* of the solubility of crystalline FEL modeled with PC-SAFT and for the glass-transition temperatures modeled with the Kwei-Equation.

System	*T_g_*		Solubility	
	*MAD* (K)	*AAD* (K)	*MAD* (K)	*AAD* (K)
FEL/Resomer^®^ R 202 S	1.13	0.65	4.75	3.76
FEL/Resomer^®^ RG 752 S	2.45	1.06	3.46	2.32
FEL/Resomer^®^ RG 502	0.97	0.39	3.51	1.42

**Table 6:** *MAD* and *AAD* of the crystalline IBU solubility modeled with PC-SAFT and for the glass-transition temperatures modeled with the Kwei-Equation.

System	*T_g_*		Solubility	
	*MAD* (K)	*AAD* (K)	*MAD* (K)	*AAD* (K)
IBU/Resomer^®^ R 202 S	8.57	4.03	1.12	0.53
IBU/Resomer^®^ RG 752 S	2.29	1.87	2.95	2.95
IBU/Resomer^®^ RG 502	1.29	1.29	1.29	1.29

**Table 7:** Characterization of PLGA copolymers modeled with PC-SAFT in this work.

Copolymer	*M_copol_*	GA Content	$z_{PLGA,LLA}$	$z_{PLGA,DLA}$	$z_{PLGA,GA}$
	(g/mol)	(mol%)			
Resomer^®^ R 202 S	13140 ^a^	0	0.5522	0.4478	-
Resomer^®^ RG 752 S	13073 ^a^	25	0.4586	0.3720	0.1694
Resomer^®^ RG 502	12880 ^a^	50	0.3426	0.2778	0.3796

^a^ Information from the supplier.

**Table 8:** Calculation of bond fractions $B_{i\alpha i\beta }$ required for modeling copolymer properties.

	LLA	DLA	GA
LLA	$=\frac{z_{PLGA,LLA}m_{PLGA}^{seg}-n_{PLGA,LLA}}{m_{PLGA}^{seg}-1}$	$=\frac{n_{PLGA,LLA}+n_{PLGA,DLA}-n_{PLGA,GA}}{m_{PLGA}^{seg}-1}$ =0 for Resomer^®^ RG 502	$=\frac{n_{PLGA,GA}}{m_{PLGA}^{seg}-1}$
DLA		$=\frac{z_{PLGA,DLA}m_{PLGA}^{seg}-n_{PLGA,DLA}}{m_{PLGA}^{seg}-1}$	$=\frac{n_{PLGA,GA}-1}{m_{PLGA}^{seg}-1}$
GA			$=\frac{z_{PLGA,GA}m_{PLGA}^{seg}-n_{PLGA,GA}}{m_{PLGA}^{seg}-1}$

**Table 9:** PC-SAFT pure component parameters used in this study.

API	${\left(\frac{m^{seg}}{M}\right)}_{\alpha }$	*M*	σ	$u/k_{B}$	$\varepsilon^{AB}/k_{B}$	$\kappa^{AB}$	$N^{assoc}$	Ref.
	(mol/g)		(Å)	(K)	(K)	(-)	(-)	
IBU	0.0122	206.28	4.432	374.651	879.415	0.03	2/2	[28]
FEL	0.0300	384.26	3.205	234.534	1581.144	0.02	2/2	(this work)
PLLA	0.0455		2.920	230	-	-	-	[41]
PDLA	0.0370		3.120	240	-	-	-	[41]
PGA	0.0313		2.860	233.9	-	-	-	[35]

## Supplementary Materials

Supplementary materials are available online.
