# Supplementary material for: Amorphous-Amorphous Phase Separation in API/Polymer Formulations
Source: Molecules. 2017 Feb 15;22(2):296. doi: 10.3390/molecules22020296 (PMC6155819; doi:10.3390/molecules22020296)
Supplement: Supplementary file 1 [file molecules-22-00296-s001.pdf]

Supplementary Materials: Amorphous-Amorphous Phase Separation in API/Polymer Formulations

Christian Luebbert, Fabian Huxoll and Gabriele Sadowski

Table S1. Measured solubility of FEL in organic solvents with different polarity.

Ethanol		Toluene		2-Propanol		Ethylacetate		Acetonitrile	
T (°C)	w_{FEL} (-)	T (°C)	w_{FEL} (-)	T (°C)	w_{FEL} (-)	T (°C)	w_{FEL} (-)	T (°C)	w_{FEL} (-)
10	0.1172	10	0.0440	10	0.0934	10	0.2132	5	0.0927
20	0.1425	20	0.0658	20	0.1212	20	0.2339	15	0.1072
30	0.2053	30	0.0983	30	0.1692	30	0.2715	25	0.1511
40	0.2736	40	0.1363	40	0.2241	40	0.3125	30	0.1615

Table S2. Binary PC-SAFT interaction parameters of FEL and organic solvents.

Solvent	k_{ij}	
Ethanol	3.47×10^{-5} T (K)	-9.69×10^{-3}
Toluene	1.78×10^{-4} T (K)	-5.06×10^{-2}
2-Propanol	4.67×10^{-5} T (K)	-8.22×10^{-3}
Ethyl acetate	2.50×10^{-4} T (K)	-8.76×10^{-2}
Acetonitrile	2.72×10^{-4} T (K)	-7.15×10^{-2}

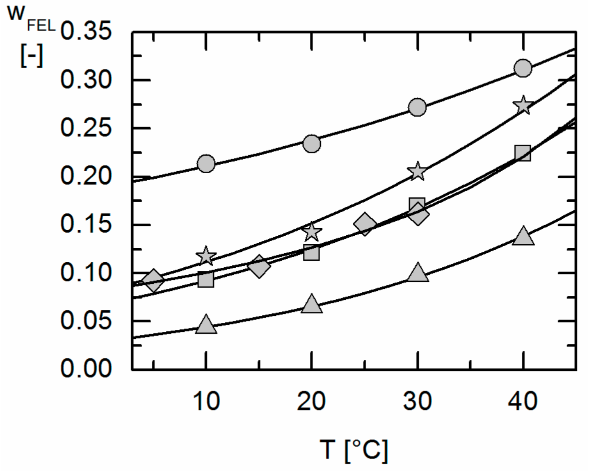

Figure S1. Solubility of FEL in different organic solvents. The symbols represent the measured solubility values for FEL in ethyl acetate (circles), ethanol (stars), acetonitrile (diamonds), 2-pronanol (squares) and toluene (triangles). The lines are the modeled solubilities with PC-SAFT.

Table S3. Solubility temperatures of FEL/PLGA formulations obtained from DSC measurements (Figure 6).

w_{FEL}	FEL/Resomer® R 202 S		FEL/Resomer® RG 752 S		FEL/Resomer® RG 502	
	Form I	Form II	Form I	Form II	Form I	Form II
0.1					-	
0.3	125.48 °C			119.86 °C	134.92 °C	
0.4	134.88 °C		134.21 °C	124.43 °C	138.65 °C	128.97 °C
0.6	136.22 °C		138.02 °C		140.38 °C	132.07 °C
0.7	139.65 °C		140.16 °C	130.90 °C	141.30 °C	
0.9	140.25 °C		141.43 °C	131.92 °C	142.87 °C	

Table S4. Solubility temperatures of IBU/PLGA formulations obtained from DSC measurements (Figure 7).

w_{IBU}	IBU/Resomer® R 202 S	IBU/Resomer® RG 752 S	IBU/Resomer® RG 502
0.1	-	-	76.20 °C
0.3	70.57 °C	73.19 °C	76.01 °C
0.4	72.72 °C	74.27 °C	76.46 °C
0.6	75.01 °C	75.71 °C	76.92 °C
0.7	75.52 °C	76.29 °C	76.98 °C
0.9	75.83 °C	76.87 °C	76.74 °C
